# Supplementary material for: Advancing Toxoplasma gondii multiplex serology
Source: Microbiol Spectr. 2024 Feb 22;12(4):e03618-23. doi: 10.1128/spectrum.03618-23 (PMC10986549; doi:10.1128/spectrum.03618-23)
Supplement: Supplemental figures — Fig. S1 to S3. [file spectrum.03618-23-s0001.docx]

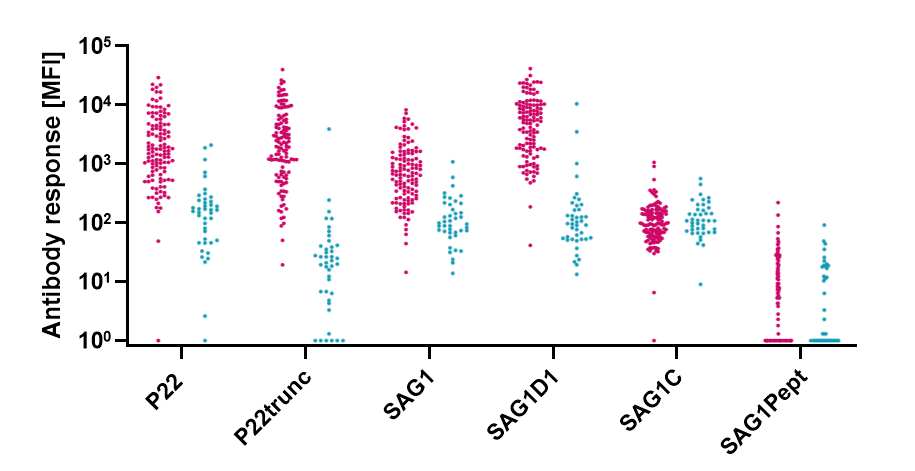


Supplementary figure 1: Antibody responses against T. gondii antigens or antigen fragments in seropositive (magenta) and seronegative individuals (blue).


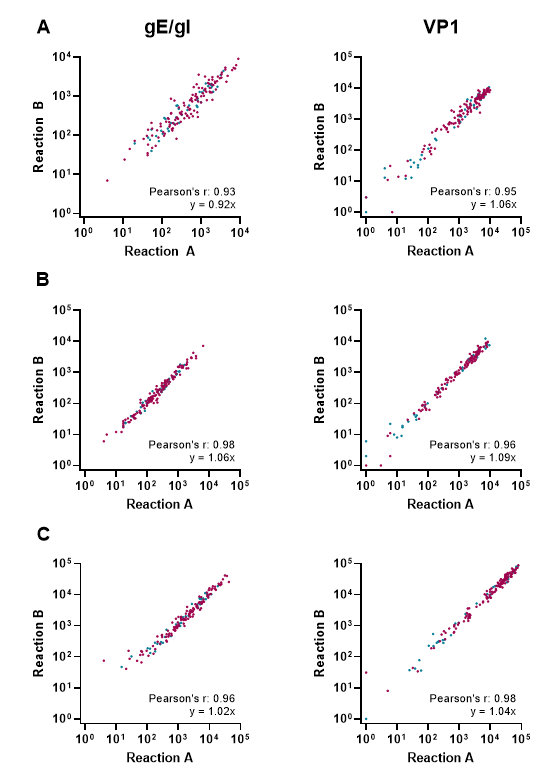


Supplementary figure 2: Antibody responses against control antigens derived from common infectious agents: envelope glycoproteins gE (ORF68) and gI (ORF67) from the Varicella zoster virus (co-loaded) and the capsid protein VP1 from the human polyomavirus 6. Two multiplex serology reactions were performed respectively to measure antibody responses against full-length and truncated T. gondii antigens, respectively, in a 1:100 (A) or a 1:1,000 (B) sample dilution. Additionally, the assay was performed on magnetic beads using a 1:1,000 sample dilution (C).

Magenta-colored dots represent individuals with a positive reference status for T. gondii, while blue dots represent individuals with a negative reference status for T. gondii. Pearson’s r was calculated to estimate the numerical correlation between the two multiplex serology reactions; the equation of the regression line was determined to estimate estimate differences between the two reactions.


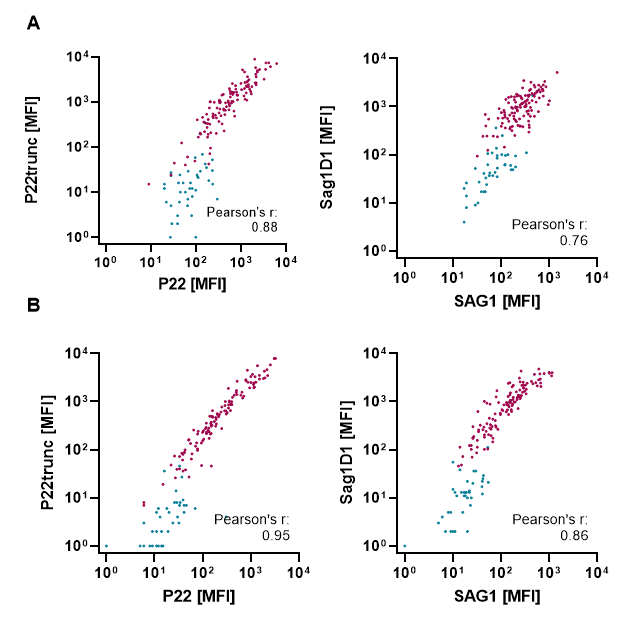


Supplementary figure 3: Antibody responses against the full-length T. gondii antigens P22 and SAG1 plotted against the respective truncated versions P22trunc and SAG1D1 in a 1:100 (A) or 1:1,000 (B) sample dilution. Pearson’s r was calculated to estimate the numerical correlation.
